# A Combination of Indoor Localization and Wearable Sensor–Based Physical Activity Recognition to Assess Older Patients Undergoing Subacute Rehabilitation: Baseline Study Results

**DOI:** 10.2196/14090

**Published:** 2019-07-10

**Authors:** Ramin Ramezani, Wenhao Zhang, Zhuoer Xie, John Shen, David Elashoff, Pamela Roberts, Annette Stanton, Michelle Eslami, Neil Wenger, Majid Sarrafzadeh, Arash Naeim

**Affiliations:** 1 Center for Smart Health University of California, Los Angeles Los Angeles, CA United States; 2 Department of Computer Science University of California, Los Angeles Los Angeles, CA United States; 3 Department of Hematology and Oncology University of California, Los Angeles Los Angeles, CA United States; 4 Department of Medicine Statistics Core, Biostatistics and Computational Biology University of California, Los Angeles Los Angeles, CA United States; 5 Department of Biomedical Sciences California School for Health Sciences Los Angeles, CA United States; 6 Department of Psychology University of California, Los Angeles Los Angeles, CA United States; 7 Rockport Healthcare Services Los Angeles, CA United States; 8 Division of General Internal Medicine University of California, Los Angeles Los Angeles, CA United States

**Keywords:** rehabilitation, frailty, remote sensing technology, wearable electronic devices, fitness trackers, monitoring ambulatory, smartwatches, bluetooth low energy beacons

## Abstract

**Background:**

Health care, in recent years, has made great leaps in integrating wireless technology into traditional models of care. The availability of ubiquitous devices such as wearable sensors has enabled researchers to collect voluminous datasets and harness them in a wide range of health care topics. One of the goals of using on-body wearable sensors has been to study and analyze human activity and functional patterns, thereby predicting harmful outcomes such as falls. It can also be used to track precise individual movements to form personalized behavioral patterns, to standardize the concept of frailty, well-being/independence, etc. Most wearable devices such as activity trackers and smartwatches are equipped with low-cost embedded sensors that can provide users with health statistics. In addition to wearable devices, Bluetooth low-energy sensors known as BLE beacons have gained traction among researchers in ambient intelligence domain. The low cost and durability of newer versions have made BLE beacons feasible gadgets to yield indoor localization data, an adjunct feature in human activity recognition. In the studies by Moatamed et al and the patent application by Ramezani et al, we introduced a generic framework (Sensing At-Risk Population) that draws on the classification of human movements using a 3-axial accelerometer and extracting indoor localization using BLE beacons, in concert.

**Objective:**

The study aimed to examine the ability of combination of physical activity and indoor location features, extracted at baseline, on a cohort of 154 rehabilitation-dwelling patients to discriminate between subacute care patients who are re-admitted to the hospital versus the patients who are able to stay in a community setting.

**Methods:**

We analyzed physical activity sensor features to assess activity time and intensity. We also analyzed activities with regard to indoor localization. Chi-square and Kruskal-Wallis tests were used to compare demographic variables and sensor feature variables in outcome groups. Random forests were used to build predictive models based on the most significant features.

**Results:**

Standing time percentage (*P*<.001, *d*=1.51), laying down time percentage (*P*<.001, *d*=1.35), resident room energy intensity (*P*<.001, *d*=1.25), resident bed energy intensity (*P*<.001, *d*=1.23), and energy percentage of active state (*P*=.001, *d*=1.24) are the 5 most statistically significant features in distinguishing outcome groups at baseline. The energy intensity of the resident room (*P*<.001, *d*=1.25) was achieved by capturing indoor localization information. Random forests revealed that the energy intensity of the resident room, as a standalone attribute, is the most sensitive parameter in the identification of outcome groups (area under the curve=0.84).

**Conclusions:**

This study demonstrates that a combination of indoor localization and physical activity tracking produces a series of features at baseline, a subset of which can better distinguish between at-risk patients that can gain independence versus the patients that are rehospitalized.

## Introduction

### Background

According to the most recent census statistics, by 2050, the population aged 65 years and older is projected to double in size to 83.7 million in the United States [1]. With the increase of this geriatric population, health care utilization will increase dramatically, with a concomitant demand for rehabilitation and in-home care after hospitalization [2]. Finding the best way to support patients during rehabilitation, both at facilities and in home, without compromising patient safety is considered to be a significant challenge. The importance of patient safety and rehabilitation has highlighted the need for constant vigilance and fostered methodologies by which patients can be remotely monitored [2-8].

Numerous studies have investigated the effectiveness of remote patient health monitoring, some suggesting the potential for such technologies to reduce the overall re-admission cost [9]. With the advent of wearable devices in recent years, remote health monitoring has evolved and drawn attention, mainly by utilizing physical activity trackers. It is widely assumed that a physical activity regimen implies behavioral patterns that can affect health outcomes. Hence, tracking these patterns and leveraging them may allow the prediction of harmful outcomes, such as falls, in a timely manner. Moreover, tracking individuals’ personalized behavioral patterns may allow for the creation of actionable messages to patients and caregivers to improve patient health and outcomes [10]. The purpose of this study was to investigate the physical activity and indoor localization features obtained from our remote patient monitoring system, Sensing At-Risk Population (SARP) [2,11-14]. This study reports on SARP sensor–based markers for rehabilitation screening within a geriatric population, exploring if SARP can be used to prospectively distinguish between at-risk patients in a subacute rehabilitation environment.

### Sensing At-Risk Population System Overview

Details of the system architecture with proximity-based sensors (beacons) and a Bluetooth-enabled smartwatch as its main components can be found in the study by Moatamed et al [2] and the patent application by Ramezani et al [14]. Building models for physical activity tracking and indoor localization was based on data collected using (1) commercially available Sony SmartWatch 3 with built-in EM7180 ± 2 g triaxial accelerometer, 420 mA battery, and BCM43340 Bluetooth module and (2) proximity beacons (MCU ARM Cortex-M4 32-bit processor with floating-point unit). To build the activity tracking and indoor localization models of SARP system, patients were consented on admission to a subacute care rehabilitation center in Los Angeles.

### Bluetooth Low Energy Beacons and Indoor Localization

**Figure 1:**
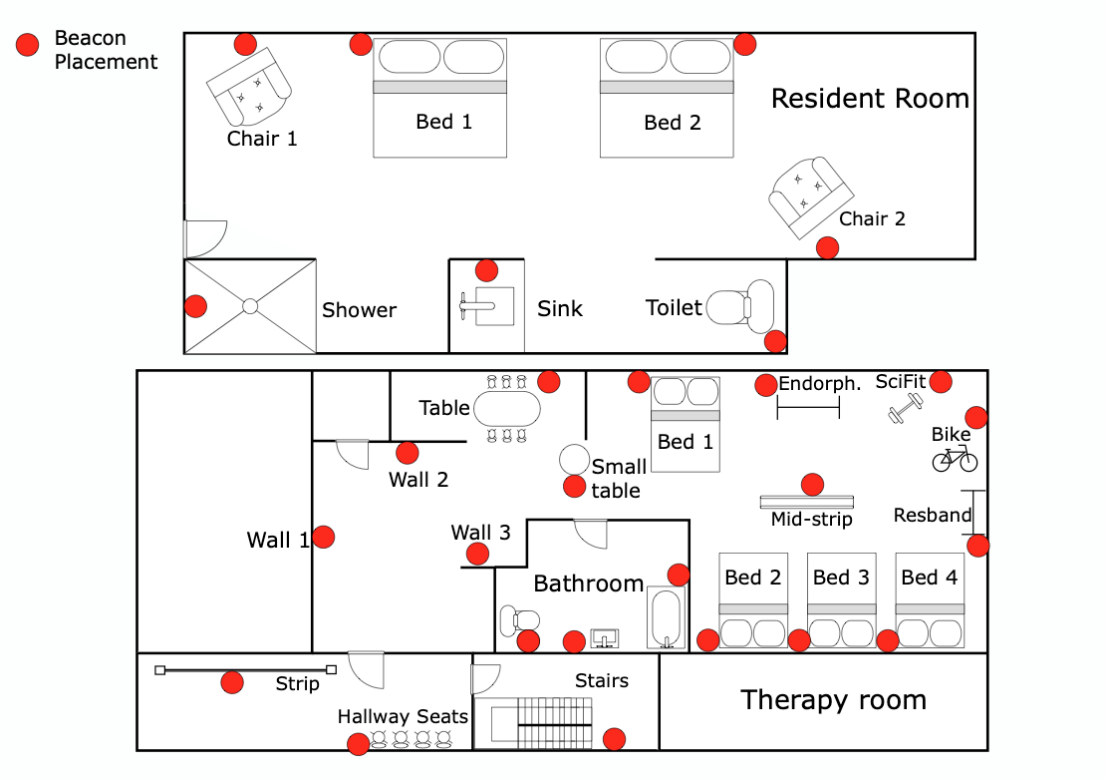
Subacute rehabilitation facility map: resident room on top and therapy room at the bottom with locations of mounted beacons shown in red.

Beacons broadcast their presence to Bluetooth-enabled devices. Utilizing the beacons’ Received Signal Strength Indicator (RSSI) values using smartwatches, the SARP system calculates the proximity of the watch to each beacon, thereby inferring the indoor location of the patient wearing that watch. BLE beacons (bluetooth low-energy sensors) have become popular in gathering contextual awareness because of durability and low cost. When used in health care, however, validating reliability and accuracy of their location information is paramount. Beacons are highly susceptible to diffraction, multipath propagation, angle-of-arrival, lack of line-of-sight, and absorption by the human body. In this project, because locations of interest were within close proximity, we considered RSSI values ranged between −50 dBm to −100 dBm. The average RSSI within the line-of-sight, measured by the watch at 1 feet distance, was −66 dBm. To achieve the best accuracy with respect to locations of interest, shown in Figure 1, considering beacons hardware specification was crucial. Beacon’s antenna configuration and the proximity of locations heavily influence the accuracy of indoor localization. Hence, to achieve a high indoor localization accuracy, it was essential to refine beacon placements iteratively. Moreover, in the rehabilitation facility shown in Figure 1, we empirically learned to set the transmission power to −12 dBm and the transmission interval to 250 ms. In studies by Bouchard et al [11-13], we proposed a few methods and considerations that can help enhance the indoor localization accuracies. A summary of the ground truth testing executed at the rehabilitation facility shown in Figure 1, with an overall accuracy >80%, can be found in a study by Moatamed et al [2].

### Accelerometer Data Processing and Physical Activity Parameters

**Figure 2:**
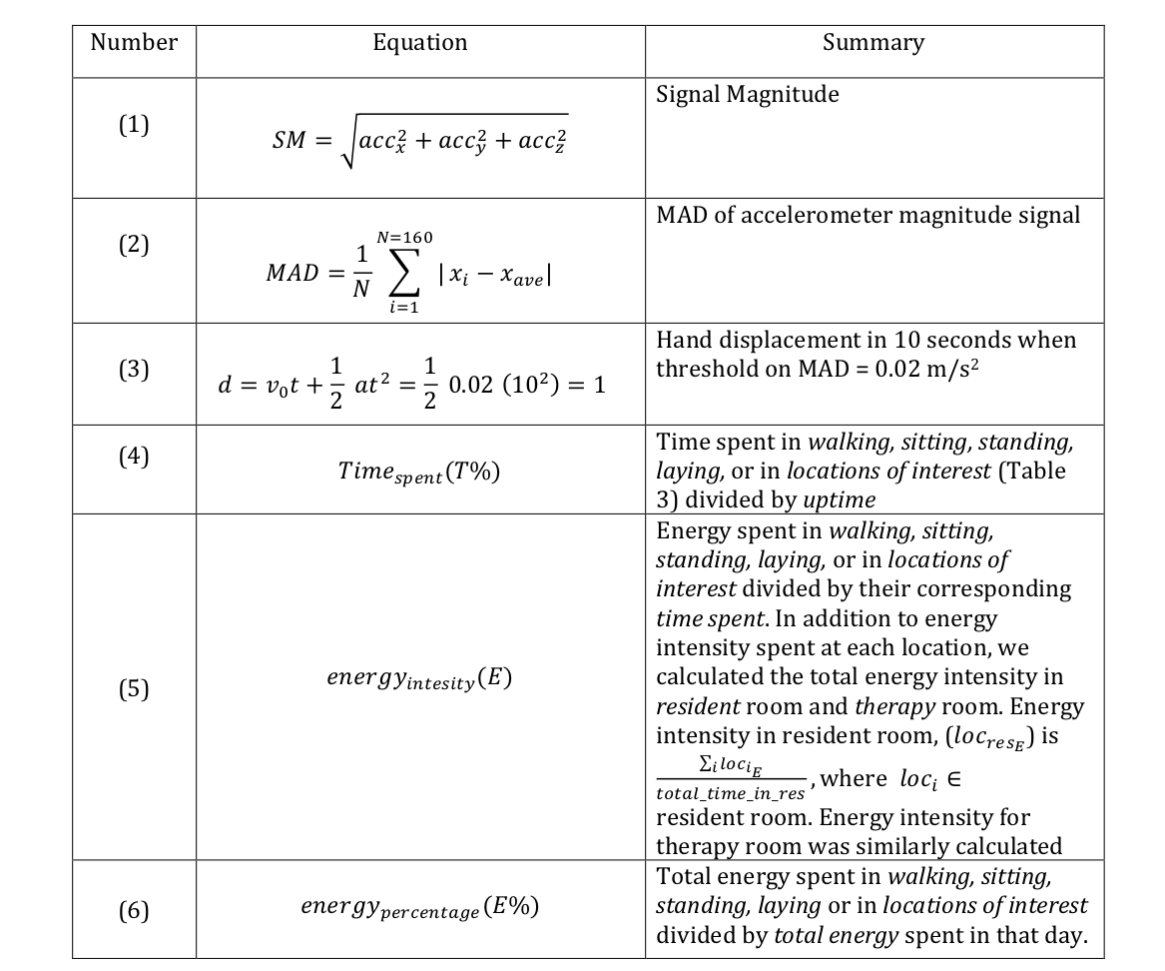
Equations. MAD: mean absolute deviation.

To infer physical activity of patients in this study, 3-axis raw acceleration signal sampled at 16 Hz was extracted, and the signal magnitude (SM) was initially calculated according to Figure 2, equation 1, where *acc* indicates acceleration force around each axis in g units including gravity (1 g=9.81 m/s^2^). The range of the acquired signal is ± 2g. Batches of 160 samples (window size of 10 seconds) were fed to a fifth order Butterworth band-pass filter with cut-off frequencies of 0.5 and 8 Hz. The filtering limited the signal to highlight the frequencies that are most representative of human motion while eliminating the direct current component. Various window sizes ranging from 4 to 12.8 seconds with different overlapping implementations have been used in different studies [15]. These characteristics are normally chosen empirically based on feature extraction, activity labeling, and other annotation factors. In this study, a window size of 10 seconds was used with a 1-second overlap [2]. After preprocessing the accelerometer data, the next step was to infer human activity (positioning) and to later translate the positioning into a quantifiable metric. However, quantifying the physical activity can be deemed challenging and will be discussed after a brief description of physical activity classification.

**Figure 3:**
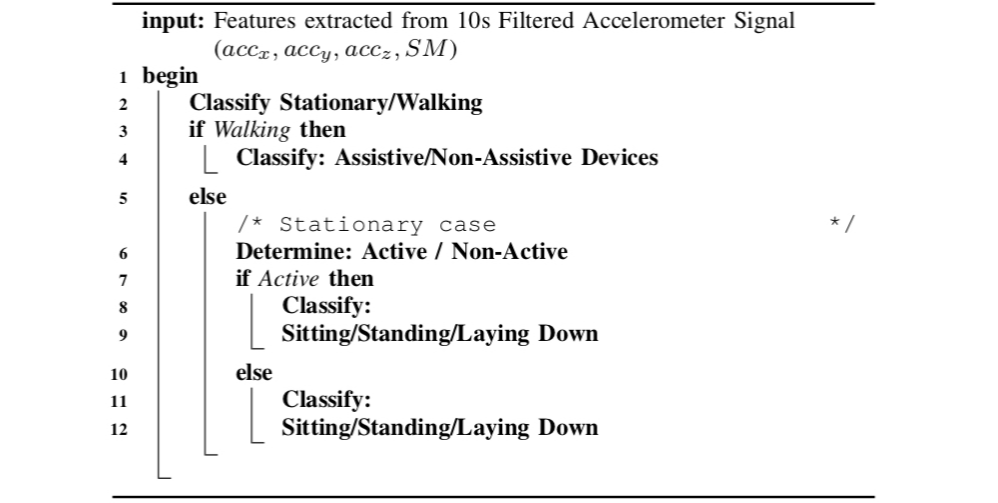
Hierarchical Activity Recognition Pseudo Code.

A decade has passed since the advent of commercially available low-cost, light-weight accelerometers. The enthusiasm about their potential in extracting physical patterns to usually, but not exclusively, improve health outcomes has led researchers to master the techniques of activity recognition [15,16]. Some researchers have even tried to infer activity intensities and predict energy consumption by comparing accelerometer patterns with measured metabolic equivalents [17-19]. Despite significant and impressive outcomes, the triumph is mostly based on analyzing small cohorts, or often a homogeneous group of people, with similar age or health conditions. Training and testing datasets in most studies are normally collated from people following a certain protocol, whereas in real life, human movements are intertwined, that is, the sequence of movements does not always form a same pattern. As such, the performance of various activity recognition algorithms/approaches applied to real-world scenarios should be taken with a grain of salt [15,16,18-20]. The following factors are influential in any human activity tracking algorithm: (1) diversity of human movement habits; (2) variety of human disabilities needing different assistive devices, yielding distinct movement patterns; (3) deficiencies of machine learning algorithms in building one-size-fits-all model; and (4) limitations to distinguish particular motions due to accelerometer placement, for instance, classifying sitting still and laying down with sensor on wrist versus waist [15,20]. To reduce the negative effect of the mentioned factors, this study uses a combination of classifications in 3 steps according to algorithm shown in Figure 3. Time and frequency domain characteristics of the signal (main, median, variance, skewness, kurtosis, peak frequency, and peak power) were used as features. SARP initially categorizes activities broadly into walking and stationary.

Walking embodies active status, and when stationary, the classifier separates brisk (active) and idle (nonactive) movements and later classifies postures into sedentary, standing, and laying down. Both Tables 1 and 2 depict the summary of physical activity (positioning) classifiers’ 10-fold cross-validation results built on 50 patients over approximately 22 hours of collated data at subacute care rehabilitation center in Los Angeles. The algorithms were later validated and refined over the course of 6 months of ground truth testing at the same skilled nursing facility.

### Step Counts Versus Raw Accelerometer Assessment

The next stage was to find a way to quantify the difference between different activity status. Step counting is a common way that has long been used to quantify the ambulatory physical activity. However, similar to activity recognition approaches explained earlier, the accuracy of step counters is often the subject of debate among researchers. Comprehensive studies with contradictory results on the accuracy of pedometers and wearable accelerometers can be found in the studies by Crouter et al [21], Mammen et al [22], and Case et al [23]. What is rather clear in using step counters/pedometers is their efficacy in quantifying *ambulatory* activities and not *stationary*. For step counters to be more accurate, a user is required to satisfy a minimum walking speed that is often mentioned in the literature as 67 m/min or even higher [24-26]. Therefore, step counters are less likely to produce accurate assessment for less mobile geriatric population. Besides, they are deemed even less effective in quantifying activities in stationary positions. Most studies assess the accuracy of step counters by asking users to walk on a treadmill, which neglects scenarios in which users are stationary, yet pedometers accumulate step counts because of movements in hand. To account for any movements (stationary and ambulatory), this study calculates mean absolute deviation (MAD) of accelerometer magnitude signal using equation 2, Figure 2. MAD calculates the statistical dispersion of acceleration from the mean and its unit is meter per second squared,

where x_i_ is the SM in each 10-second window, and the x_ave_ is the average of accelerometer magnitude for 160 samples (10-second epoch×16 Hz). MAD of accelerometer magnitude represents the average magnitude of acceleration within an interval (in this case, 10 seconds) and is proportionate to force applied to the watch by patient since f=ma. This value multiplied into displacement will produce relative work and energy. Take into account that calculating displacement from acceleration, however, is not very accurate because it is the result of accelerometer’s double integration, that is, any acceleration jitter accumulates and yields big drifts in displacement. Calculating force, however, is accurate and proportionate to energy; hence, the term *energy* has been used in this study to quantify human activity movements.

Another way of quantifying activity is to integrate each acceleration channel to produce kinetic energy using e=1/2m.v^2^. This way, however, requires more calculations compared with MAD; for the actual speed, each channel should be considered separately so that the direction of acceleration and deceleration that are removed in SM will be taken into account.

It is worth highlighting that by using a smartwatch accelerometer, it is only possible to calculate the force, proportionate to energy, that is spent *on the watch*. Hence, if a patient is carrying a weight on the watch-worn hand, the energy expenditure of the patient will not change with regard to the watch.

Active/nonactive is determined in this study using an empirical threshold of 0.02 m/s^2^ (2 cm/s^2^) over the MAD value. As explained earlier, calculating displacement from the accelerometer is not highly reliable. However, for illustrative purposes, assume the initial speed of hand movement in each window of 10 seconds is zero. Using equation 3 shown in Figure 2, the value 0.02 indicates that a patient’s hand displacement has been 1 m in 10 seconds. In case of equal or greater shifts, the patient is considered active, otherwise, idle (nonactive).

**Figure 4:**
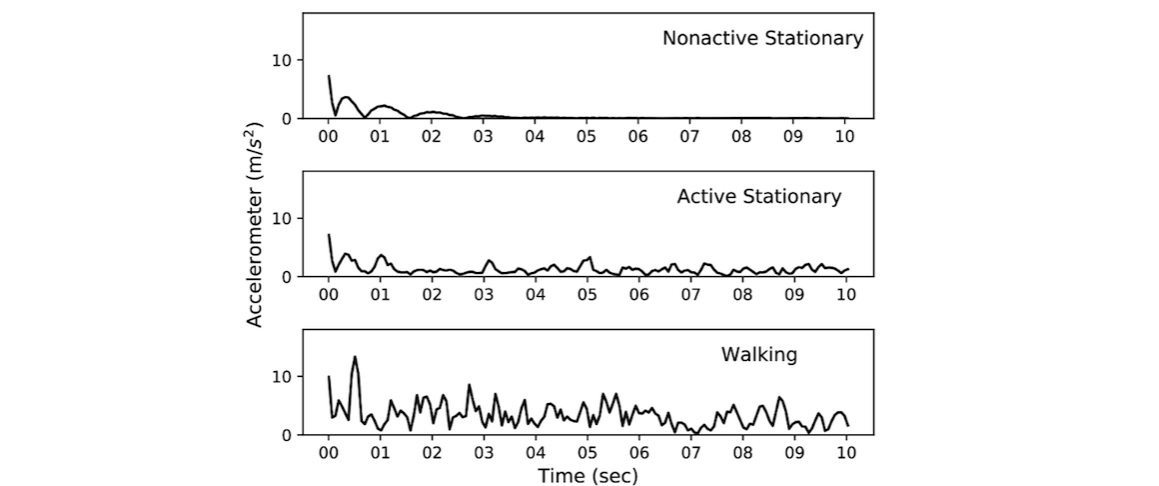
Magnitude of accelerometer signal after filtering (direct current component removed before filtering).

Figure 4 shows 10-second examples of acceleration SM of a person. It illustrates the difference in walking, active and nonactive stationary positions.

**Table 1:** Online watch classifier.

Class	TP^a^ rate	FP^b^ rate	Precision	Recall	F-measure	ROC^c^ area
Stationary	0.992	0.015	0.977	0.992	0.984	0.954
Walking	0.985	0.008	0.995	0.985	0.990	0.992
Weighted average	0.988	0.011	0.988	0.988	0.974	0.929

^a^TP: true positive.

^b^FP: false positive.

^c^ROC: receiver operating characteristic.

**Table 2:** Activity recognition: positioning.

Position	Accuracy	Precision	Recall	F-measure
Stand	91	0.94	0.91	0.92
Sit	93.7	0.87	0.93	0.90
Lay	90.8	0.97	0.90	0.94
Walk	95.1	0.92	0.95	0.94

## Methods

### Overview

From June 2016 to November 2017, we recruited patients after admission to a subacute rehabilitation center in Los Angeles. We performed a cross-sectional baseline study of this cohort to better understand data features collected by the SARP system. We investigated the prevalence of physical activity tracking features and indoor localization features at baseline for both outcome groups (hospital vs long-term care). Moreover, we assessed their efficacy in determining the outcome (hospital vs long-term care).

### Participants

Participants aged older than 60 years were recruited from a subacute rehabilitation facility in Los Angeles. The study cohort contains patients who had been admitted to a subacute rehabilitation center for 21 days. After this period, patients were either re-admitted to hospital (H) or stayed in community (C; either at home or long-term care). The inclusion criteria were broad, allowing any patient to participate as long as they were aged older than 60 years, English speaking, and able to consent with the exclusion criteria including movement disorders or paralysis of the upper or lower extremity. The diversity of cohort included patients who were a postsurgical, poststroke, and postclinical decompensation because of medical illnesses. Eligible participants signed a consent form approved by the University of California, Los Angeles, Institutional Review Board.

### Study Design

Patients were given a smartwatch by a clinical coordinator every morning at 9 am. Patients were asked to wear their watches at all times until the coordinator collected the watch at around 6 pm every day. Watch batteries were expected to last longer than the protocol period (>9 hours). Patients normally stayed in the *resident room* (bedroom) and were scheduled for an hour of daily exercise and activity in the *therapy* room. Beacons were mounted at *locations of interest* (Table 3), shown with color dots in Figure 1 within bedroom and therapy room. Take into account that despite imposing an identical protocol for all patients, daily collected data from each individual may differ. This is primarily because of patients not complying with the protocol at all times, losing interest during the day, feeling uncomfortable, and getting concerned about their privacy. Therefore, to provide a situation in which a fair comparison among patients can be enforced, we determined *analysis inclusion criteria*.

**Table 3:** Locations of interest. For sensor-based feature assessment throughout the paper, shower, toilet, and sink are considered as bathroom; walls 1, 2, and 3 as wall; beds 1 to 4 inside the therapy room and beds 1 and 2 inside the resident room as beds.

Location	Sublocations
Resident room	Bed, chair, shower, toilet
Therapy room	Bed, resband, bike, endorphine, strip, table, small table, hallway, seats, wall, hallway doors, sink, bath

### Analysis Inclusion Criteria

For this baseline analysis, we included study participants who satisfied the following constraints: (1) patients with 4 hours or more of watch wear time data in at least 1 day within the first 3 days of admission (*defined as baseline*); and (2) having 15 min or more of therapy room wear time in that particular *baseline* day. In case both inclusion criteria were satisfied on more than 1 day, the earliest day was selected as *baseline*. The reason for choosing 4 hours or more wear time was to set a standard minimum; given the health of this population whom mostly recently discharged from the hospital, we anticipated variability in watch usage. To have a minimum standard, we agreed that patients needed to wear the watch more than 50% of the available hours per day (in this study, 8 hours).

The hours when the watch was not worn were excluded from the study; therefore, baseline hours may not be *consecutive*.

### Measures

#### Demographic and Clinical Characteristics

We collected the demographic characteristics of patients such as age, race, gender, and ethnicity. We also translated the clinical coordinator’s assessments including usage of assistive devices and their type, measures of activity of daily living (ADL), pain (yes/no), and number of active diagnosis (more or less than 10). We investigated the significance of such characteristics in distinguishing the outcome (community vs hospital).

#### Sensor-Based Parameters

Sensor-based features are combination of 3 groups of parameters that are achieved by harnessing smartwatch and BLE beacons. The features are based on (1) activity recognition such as sitting time and standing time; (2) indoor localization, for example, time in bed, time in bathroom, or therapy room; and (3) row acceleration quantification, MAD (energy; see section Sensing At-Risk Population System Overview). By combining these attributes, we achieved features such as sitting time in bed or energy spent in walking or in bed.

To perform a fair comparison among patients with different watch wear time, we *normalized* features: time spent (minutes) in a certain physical activity or location was divided by *uptime* (the total watch wear time in a day in minutes) to yield normalized time features. Uptime is an essential factor in providing fair comparison

We investigated the significance of sensor-based features with respect to the outcomes: hospital versus community. All measurements are at baseline, that is, the day that satisfies inclusion criteria from 9 am to 6 pm. We calculated “time spent in percentage,” “energy intensity (E),” and “energy spent in percentages,” as shown in equations 4, 5, and 6 in Figure 2.

To recap, for each individual, *time-related* features such as *sitting time* were divided by *uptime*. Energy-related features such as *walking* were divided by: (1) the uptime, yielding energy intensity and (2) their total daily value, producing the energy percentage.

### Statistical Analysis

We explored the capability of baseline sensor-based and demographic features to distinguish between subacute rehabilitation patients based on their outcomes (ie, re-admitted to hospital (H) vs staying in the community (C) either long-term care or home). Chi-squared tests were used to compare categorical demographic variables between outcome groups. We compared quantitative demographic variables and sensor-based metrics (physical activity derived from watch accelerometer and indoor localization inferred from BLE beacons RSSI) between groups using the Kruskal-Wallis test. Cohen *d* was used to summarize the effect size and illustrate the discriminatory power of each feature. Commonly, 0.2, 0.5, and 0.8 are Cohen *d* cut-off values indicating small, medium, and large effect size, respectively. Spearman rho was used to measure correlations between physical activity and location-based features.

#### Predictive Models of Outcome

We investigated the capability of features at baseline to triage and predict patients who were re-admitted to the hospital or who stayed in community. We built random forest models (maximum depth=2, random state=40, and class_weight=balanced), with hospital patients as positive group. We used single or combination of features with highest statistical significance in distinguishing outcomes according to Kruskal-Wallis tests. Model generation and evaluating performance characteristics (3-fold cross-validation) including sensitivity, specificity, accuracy, and area under the curve (AUC) estimation were performed using Python Programming Language libraries Pandas (version 0.21.0) and Numpy (version 1.14.5), Scipy (version 1.0.0), and Scikit-learn (version 0.19.1) [27-30].

## Results

### Demographic and Clinical Characteristics

From 184 consented subjects, 30 were excluded because of not satisfying the analysis inclusion criteria. A total of 154 patients were included in this study in which 145 (94.2%) of subjects discharged home/community (C), and 9 (5.8%) re-admitted to hospital (H) at the end of their rehabilitation process. Table 4 presents detailed sociodemographic and clinical characteristics of this cohort, such as age, gender, race-ethnicity, presence of pain, number of active diagnoses, usage of assistive devices, and ADL. Table 4 indicates the mean (SD) and number of patients included for every particular parameter. Among the clinical assessments, Table 4 shows that ADL Toilet is significant in determining the outcome (*P*=.007) with 65% of the cohort in need of extensive assistance and 35% limited assistance.

**Table 4:** Sociodemographic and clinical characteristics of the cohort of 154 patients.

Parameter		Community	Hospital	Community vs hospital (*P* value)
Subjects, n (%)		145 (94.2)	9 (5.8)	—^a^
Age (years), mean (SD)		82.16 (9.55)	84.22 (13.87)	.24
**Gender, n (%)**				.56
	Female	104 (71.7)	4 (44.4)	
	Male	41 (28.3)	5 (55.6)	
**Race/ethnicity, n (%)**				>.99
	Asian	5 (3.4)	0 (0.0)	
	Black/African American	14 (9.7)	0 (0.0)	
	Hispanic/Latino	4 (2.7)	0 (0.0)	
	Native/Hawaiian Pacific Islander	3 (2.1)	0 (0.0)	
	White	119 (82.1)	9 (100)	
**Pain present, n (%)**				.92
	No	44 (31.7)	1 (14.3)	
	Yes	95 (68.3)	6 (85.7)	
**Active diagnoses, n (%)**				>.99
	<10	22 (15.2)	1 (11.1)	
	≥10	123 (84.8)	8 (88.9)	
**ADL^b^** **transfer, n (%)**				.77
	Limited assistance	65 (45.1)	2 (22.2)	
	Extensive assistance	79 (54.9)	7 (77.8)	
**ADL dress, n (%)**				.96
	Limited assistance	32 (22.2)	1 (11.1)	
	Extensive assistance	112 (77.8)	8 (88.9)	
**ADL eat, n (%)**				.91
	Independent	128 (88.9)	7 (77.8)	
	Supervision	4 (2.8)	0 (0.0)	
	Limited assistance	9 (6.2)	1 (11.1)	
	Extensive assistance	3 (2.1)	1 (11.1)	
**ADL toilet, n (%)^c^**				.007
	Limited assistance	50 (34.7)	1 (11.1)	
	Extensive assistance	94 (65.3)	7 (77.8)	
	Total dependence	0 (0.0)	1 (11.1)	
**ADL walk room, n (%)**				.73
	Limited assistance	73 (50.7)	2 (22.2)	
	Extensive assistance	59 (41.0)	5 (55.6)	
	Activity did not occur	12 (8.3)	2 (22.2)	
**ADL walk hall, n (%)**				.88
	Limited assistance	73 (50.7)	2 (22.2)	
	Extensive assistance	64 (44.4)	6 (66.7)	
	Activity occurred only once or twice	2 (1.4)	0 (0.0)	
	Activity did not occur	5 (3.5)	1 (11.1)	
**ADL walk on unit, n (%)**				.85
	Supervision	1 (0.7)	0 (0.0)	
	Limited assistance	71 (49.3)	2 (22.2)	
	Extensive assistance	72 (50.0)	7 (77.8)	
**ADL hygiene, n (%)**				.84
	Supervision	2 (1.4)	0 (0.0)	
	Limited assistance	71 (49.3)	2 (22.2)	
	Extensive assistance	71 (49.3)	7 (77.8)	
**ADL bed, n (%)**				.61
	Supervision	1 (0.7)	0 (0.0)	
	Limited assistance	83 (57.6)	2 (22.2)	
	Extensive assistance	60 (41.7)	7 (77.8)	
**Urinary continence, n (%)**				.09
	Always continent	117 (81.2)	4 (44.4)	
	Occasionally incontinent	4 (2.8)	0 (0.0)	
	Frequently incontinent	8 (5.6)	2 (22.2)	
	Always incontinent	7 (4.8)	3 (33.3)	
	Not rated	8 (5.6)	0 (0.0)	
**Bowel continence, n (%)**				.08
	Always continent	128 (88.9)	5 (55.6)	
	Occasionally incontinent	3 (2.1)	0 (0.0)	
	Frequently incontinent	7 (4.8)	1 (11.1)	
	Always incontinent	6 (4.2)	3 (33.3)	
**Assistive devices, n (%)**				.97
	Walker	1 (0.7)	0 (0.0)	
	Wheelchair	5 (4.0)	1 (14.3)	
	Walker and wheelchair	123 (94.6)	6 (85.7)	
	Cane and wheelchair	1 (0.7)	0 (0.0)	

^a^Not applicable.

^b^ADL: activity daily living.

^c^Parameters with *P*<.05.

### Energy Intensity Features Assessment

Amongst sensory-based features shown in Figure 2, equations (4-6), energy intensity features are the ratio of the total energy spent in a particular activity or location to their corresponding time spent. Taking into account, indoor localization capability of SARP system enabled us to calculate the energy spent at each location of interest, sum of which was broadly categorized into (1) energy intensity in resident room and (2) energy intensity in therapy room. According to Table 5, energy features that best discriminated community and hospital patients were energy intensity in resident room (*P*<.001, *d*=1.21), resident_bed (*P*<.001, *d*=1.23), resident_bath (*P*=.004, *d*=1.18), and total energy intensity (*P*=.003, *d*=0.87). Features such as energy intensity of laying down (*P*=.02), and therapy_bathroom (*P*=.02), despite statistical significance, have low effect sizes (*d*=0.418 and *d*=0.17, respectively). Moreover, with *P*<.001 and *d*=1.25, energy intensity in resident room has high discriminatory power with respect to outcome.

**Table 5:** Sensor-based (activity and indoor localization) features: assessment according to outcomes.

Feature		Community, mean (SD)	Hospital, mean (SD)	*P* value	Effect size^a^	Frequency (n)		
						Community		Hospital
**Energy % parameters**								
	Active^b^	2.37 (3.84)	1.00 (1.29)	.001	1.24	145	9	
	Walking	2.37 (3.84)	1.00 (1.29)	.08	0.50	145	9	
	Standing^b^	59.70 (8.70)	57.92 (6.39)	.002	1.24	145	9	
	Sitting^b^	17.83 (9.69)	13.33 (8.90)	.02	0.86	145	9	
	Laying down^b^	20.10 (6.43)	27.73 (9.94)	.04	0.54	145	9	
**Energy intensity parameters**								
	Total energy^b^	52.61 (18.23)	35.85 (16.53)	.003	0.87	145	9	
	Active	11.94 (18.27)	6.05 (8.02)	.30	0.42	145	9	
	Walking	450.47 (253.08)	366.45 (218.66)	.44	0.34	145	9	
	Standing	85.93 (26.92)	82.27 (36.12)	.32	0.11	145	9	
	Sitting	184.33 (97.58)	156.19 (104.74)	.31	0.28	145	9	
	Laying down^b^	26.23 (8.68)	19.54 (7.35)	.02	0.418	145	9	
**Energy intensity—therapy room**								
	Energy therapy room	70.75 (43.11)	68.49 (63.56)	.36	0.04	145	9	
	Bathroom^b^	74.84 (49.02)	62.35 (83.54)	.02	0.17	114	8	
	Strip	57.84 (42.33)	13.03 (8.30)	.06	1.43	88	2	
	Bed	60.22 (40.27)	39.09 (7.15)	.27	0.72	97	4	
	Resband	61.06 (43.10)	75.73 (85.49)	.57	0.20	100	6	
	Bike	91.80 (76.82)	120.58 (38.41)	.31	0.43	36	2	
	Scifit	98.39 (55.04)	0.0 (0.0)	—^c^	—	14	0	
	Endor	41.38 (6.74)	0.0 (0.0)	—	—	3	0	
	Midstrip	56.46 (48.92)	65.46 (24.53)	.38	0.22	45	3	
	Small table	61.07 (40.37)	148.47 (138.78)	.53	0.71	57	3	
	Table	93.49 (66.75)	0.0 (0.0)	—	—	56	0	
	Hallway seats	42.58 (43.13)	32.52 (7.89)	.87	0.32	43	3	
	Stairs	133.48 (128.07)	0.0 (0.0)	—	—	8	0	
	Wall	57.07 (28.49)	25.61 (0.0)	.17	—	73	1	
**Energy intensity—resident room**								
	Energy resident room^b^	43.32 (17.44)	26.99 (6.05)	<.001	1.25	145	9	
	Bed^b^	43.93 (19.01)	25.76 (4.37)	<.001	1.23	144	9	
	Bathroom^b^	55.89 (27.95)	32.50 (9.30)	.004	1.18	141	9	
	Chair	42.45 (20.61)	0.0 (0.0)	—	—	5	0	
**Time % parameters**								
	Active^b^	12.92 (6.52)	6.94 (4.01)	.001	1.10	145	9	
	Walking	0.35 (0.51)	0.15 (0.27)	.09	0.44	145	9	
	Standing^b^	44.22 (7.94)	32.68 (7.30)	<.001	1.51	145	9	
	Sitting^b^	8.60 (8.36)	6.16 (7.36)	.04	0.31	145	9	
	Laying down^b^	46.83 (9.83)	60.99 (11.11)	<.001	1.35	145	9	
**Time spent %—therapy room**								
	Bathroom	0.03 (0.04)	0.06 (0.08)	.16	0.27	114	8	
	Strip	0.01 (0.03)	0.005 (0.002)	.62	0.48	88	2	
	Bed	0.62 (0.19)	0.55 (0.23)	.64	0.43	97	4	
	Resband^b^	0.02 (0.02)	0.05 (0.03)	.03	0.74	100	6	
	Bike	0.03 (0.03)	0.01 (0.002)	.51	0.80	36	2	
	Scifit	0.03 (0.02)	0.0 (0.0)	—	—	14	0	
	Endor	0.009 (0.01)	0.0 (0.0)	—	—	3	0	
	Midstrip	0.02 (0.02)	0.02 (0.02)	.31	0.49	45	3	
	Small table^b^	0.02 (0.03)	0.04 (0.02)	.04	0.50	57	3	
	Table	0.06 (0.05)	0.0 (0.0)	—	—	56	0	
	Hallway seats	0.006 (0.004)	0.01 (0.16)	.64	0.78	43	3	
	Stairs	0.02 (0.04)	0.0 (0.0)	—	—	8	0	
	Wall	0.01 (0.02)	0.01 (0.0)	.98	—	73	1	
**Time spent %—resident room**								
	Bed	0.62 (0.19)	0.55 (0.23)	.16	0.12	144	9	
	Bathroom	0.21 (0.17)	0.25 (0.20)	.92	0.52	141	9	
	Chair	0.007 (0.03)	0.0 (0.0)	—	—	5	0	

^a^Effect sizes have been calculated as Cohen *d*.

^b^Parameters with *P*<.05.

^c^Not applicable (the *P* value or effect size cannot be calculated).

**Figure 5:**
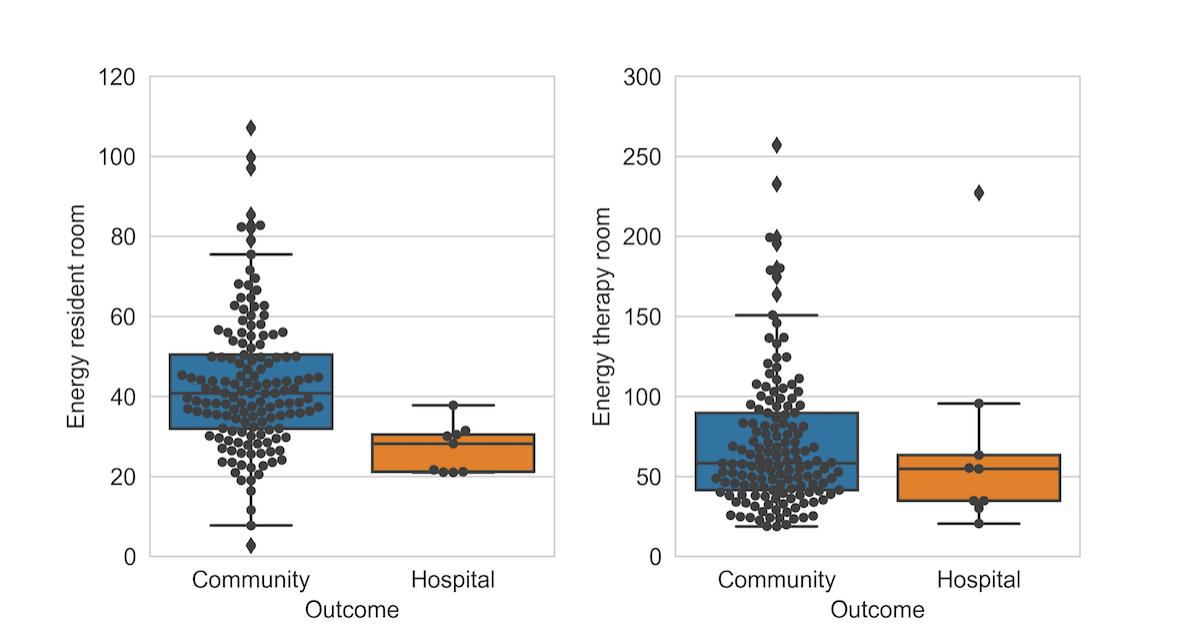
Energy intensity distribution.

Figure 5 depicts the energy intensity distributions between 2 groups in resident and therapy rooms. It shows that energy intensity in therapy room in both groups has similar mean value (line within the box); therefore, a clear distinction cannot be made within 2 groups based on that feature. However, the mean value of community group in resident room is clearly higher than in hospital patients.

**Figure 6:**
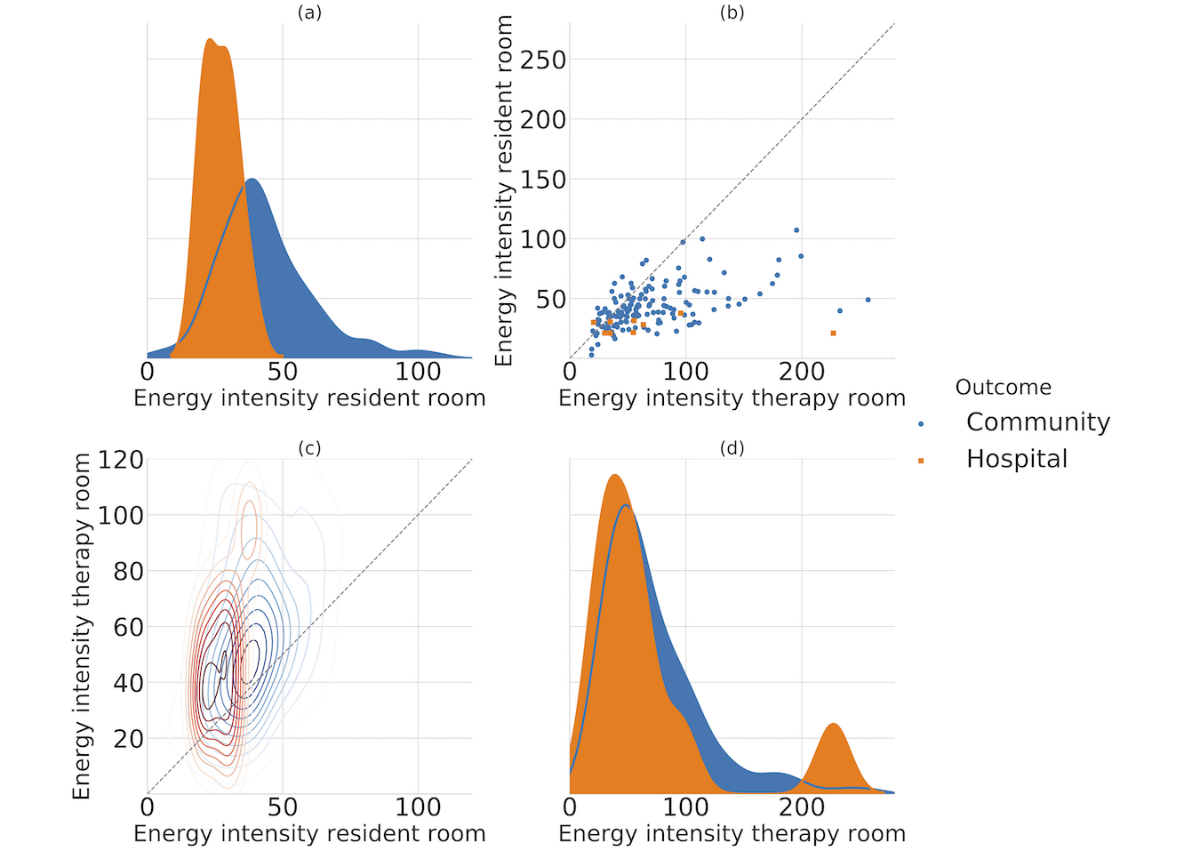
Gauging energy intensity in community versus hospital.

**Figure 7:**
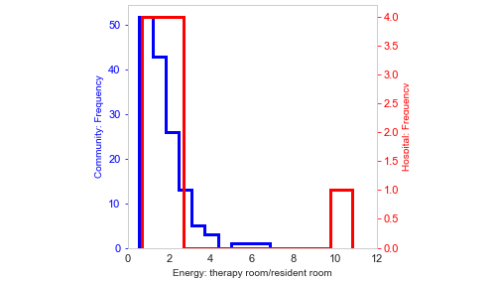
Distribution of patients spending energy in therapy room compared with resident room. X-axis indicates the ratio of energy in therapy to resident room.

**Figure 8:**
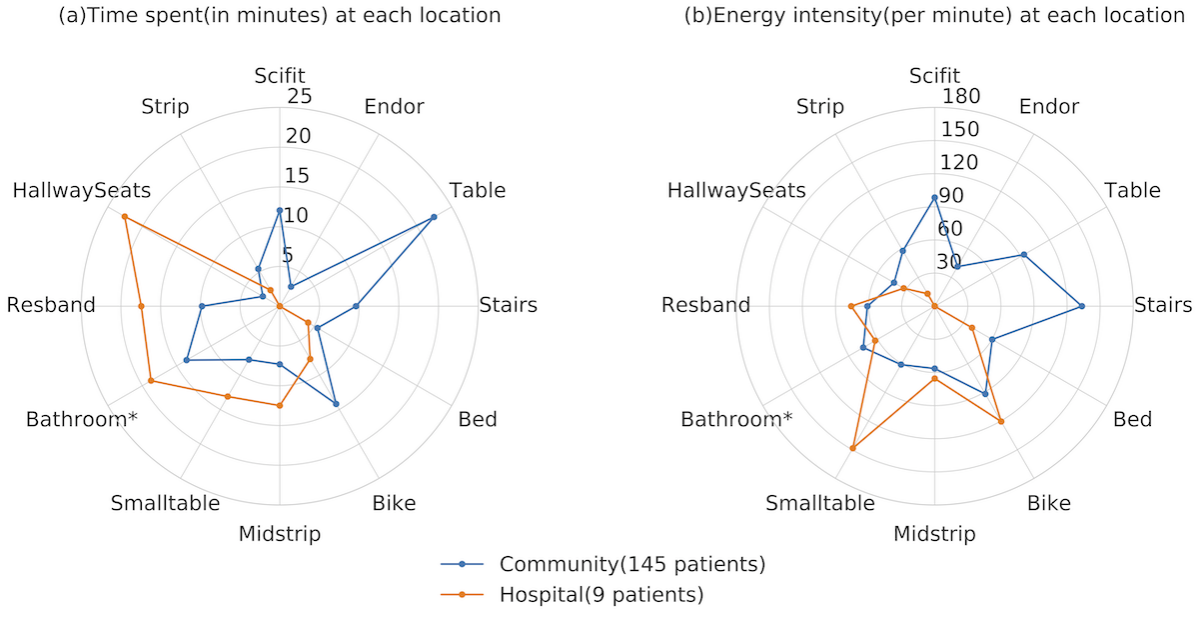
Time and energy intensity details of therapy room.

Kernel density estimation (KDE) distributions are shown in Figure 6 (subplots A and D)*.* The figure attests to the distinction of energy intensity in resident room among community and hospital patients (subplot A). However, the KDE of energy intensity in therapy room (subplot D) does not indicate the same discriminatory power. Figure 6 (subplot B) indicates that energy intensity of most patients in therapy room is higher compared with resident room for both outcome groups because most patients fall below the identity line. Points shown on the identity line represent patients with same therapy and resident intensities. According to subplot (C), the center core of the contour plot (representing most patients) in community group is almost circular contrary to hospital patients. This indicates that the ratio of resident to therapy intensity is closer to one (ie, same activity intensities). On the contrary, more oval shape of the contour core in hospital group can imply that most patients are persistently more active during therapy sessions while being less active in their resident room. The increase in energy levels can be seen clearly in Figure 7. The figure depicts the ratio of energy intensity in therapy room to resident room. Most patients in hospital outcome group, demarcated by red line, fall around number 2. In other words, therapy room energy intensity is twice the resident room for most patients in hospital group. However, 50 patients in community group (blue histogram) have the ratio close to 1, that is, the same intensity in both therapy and resident room. A more detailed scenario of both groups within the therapy room can be found in Figure 8 and Table 6.

Average time spent and energy intensity at each therapy location stratified by groups are shown in Figure 8. It is clear that hospital group spent *no time* at stairs, scifit, table, and endorphine. The 5 most intensive activities were small table, *stairs, scifit, table, and bike*. Small table and table are places where patient normally carried out hand pedaling exercises. Table 6 further highlights the details of therapy room location/facility usage in each group. More than 70% of participants from both groups had used *bed* and *bathroom* in therapy room, with *bathroom* ’s *P*<.05 (Table 5). However, it is worth mentioning that the effect size of *bathroom* energy intensity is small: 0.17 (cut-off regions: 0.2 small, 0.5 medium, and 0.8 large). Furthermore, Figure 8 reveals that both groups’ intensities at bed and bathroom were less than 60 per min. In a study by Razjouyan et al [8], a cutoff point of 90 is suggested to differentiate between light and moderate-to-vigorous activities.

**Table 6:** Frequency of therapy room location/facility usage by group.

Location/facility	Frequency of facility usage	
	Community, n (%)	Hospital, n (%)
Scifit	14 (9.6)	0 (0.0)
Endor	3 (2.1)	0 (0.0)
Table	56 (38.6)	0 (0.0)
Stairs	8 (5.5)	0 (0.0)
Bed	118 (81.4)	7 (77.8)
Bike	36 (24.8)	2 (22.2)
Midstrip	45 (31.0)	3 (33.3)
Small table	57 (39.3)	3 (33.3)
Bathroom^a^	114 (78.6)	9 (100.0)
Resband	100 (69.0)	7 (77.8)
Hallway seat	43 (29.7)	3 (33.3)
Strip	88 (60.7)	3 (33.3)

^a^Parameters with *P*<.05.

**Figure 9:**
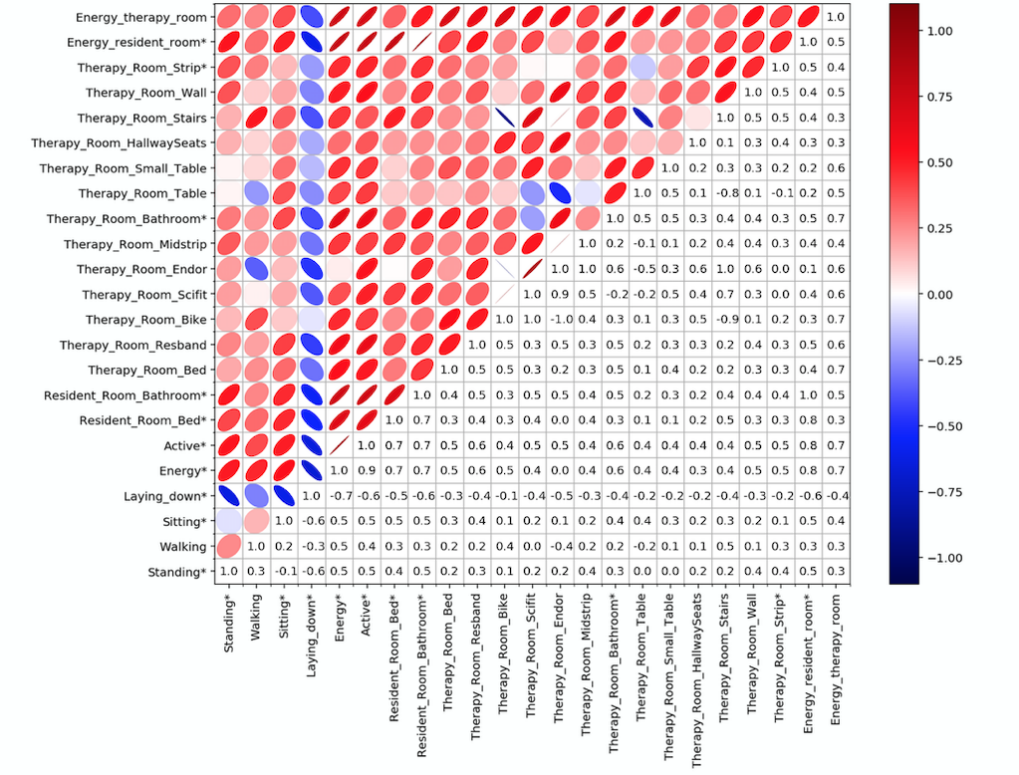
Correlations among sensor-based features. Asterisk indicates parameters with *P*<.05.

Figure 9 illustrates Spearman correlations among features. According to annotations explained in the Features section, E indicates energy intensity, E% denotes energy percentage, and T% shows the percentage of time spent. Circles, contrary to ovals, correspond to low correlation, whereas lines imply the highest correlation. Darker spectrum on either side (red or blue) represents higher correlation; red implies positive, whereas blue is indicative of negative correlation. It is clear from the figure that laying down is negatively correlated with the rest of the features. Bath and bed in resident room are understandably correlated strongly with energy spent in resident room because almost all activities happened in those 2 locations, and patients hardly used the chair. Bed, bath, resband, small table, bike, and scifit are strongly correlated with energy spent in therapy room. It is clear that being active is highly correlated with overall energy intensity. Resident room energy intensity is strongly correlated with overall energy intensity.

### Energy Percentage Features Assessment

Energy percentage feature, as mentioned in Figure 2, is the percentage of energy spent in *walking, sitting, standing, laying*, or energy spent in *locations of interest* divided by *total energy* spent in that day. According to Table 5, community patients are more active (*P*=.001, *d*=1.24) than patients re-admitted to the hospital. Meanwhile, energy percentage of standing (*P*=.002, *d*=1.24) and sitting (*P*=.02, *d*=0.86) of the community group is higher than those in hospital group. Other than walking, all energy percentage parameters were shown significant in distinguishing between both groups. Walking is not significant in distinguishing the outcome: Energy (%) in walking (*P*=.08, *d*=0.50) and energy intensity during walking (*P*=.44, *d*=0.34).

### Time Features Assessment

According to Table 5, standing time (%) has the strongest discriminatory power (*P<*.001, *d*=1.51) among all watch-derived parameters. Community group has higher time percentage in laying down (*P*<.001, *d*=1.35) and active state (*P*=.001, *d*=1.24) compared with hospital group. Despite statistical significance of sitting time (%), its effect size is between small and medium (*P*=.04, *d*=0.31). Walking time was quite negligible (<1% of time for both groups with *P*=.09, *d*=0.44), whereas overall active state, which captures walking and stationary active periods, was highly significant (*P*=.001, *d*=1.10). As shown in the table, none of the time (%) parameters in resident room have the ability to discriminate between the 2 outcome groups.

### Performance of Predictive Models at Baseline

Random forest models were built based on the most statistically significant features. In reviewing Table 4, the top 3 most influential features in distinguishing the outcomes were % standing time (*P*<.001, *d*=1.51), % laying down time (*P*<.001, *d*=1.35), *and resident room energy intensity (P*<.001, *d*=1.25). Results of 3-fold cross-validation models with their corresponding AUC score are presented in Table 7. Take into account that the sensitivity (recall) presented in the table is not the weighted average and reflects only recall of minority (H) group. Specificity indicates the true negative rate when negative group is comprised most patients returning to community setting (C) after the rehabilitation period.

**Table 7:** Predictive models: 3-fold cross-validation (community, n=48; hospital, n=3).

Features	Sensitivity, mean (SD)^a^	Specificity, mean (SD)^a^	Accuracy, mean (SD)^a^	AUC^b^, mean (SD)
Standing time (%)	22.2 (31.4)	74.4 (15.3)	71.4 (12.9)	0.62 (0.06)
Standing time (%), laying down time (%)	11.1 (15.7)	91.0 (0.9)	86.4 (1.5)	0.70 (0.10)
Standing time (%), laying down time (%), resident room energy intensity (%)	44.4 (41.6)	87.6 (4.3)	85.1 (5.5)	0.85 (0.09)
Resident room energy intensity	77.7 (15.7)	74.5 (8.5)	74.7 (7.3)	0.84 (0.10)

^a^Mean (SD) reported for the validation datasets based on a 3-fold cross-validation. Mean and SD are calculated across all 3 folds.

^b^AUC: area under the curve.

## Discussion

To our knowledge, this is the first study that has combined indoor localization and accelerometer-based physical activity recognition to assess older patients. A subset of indoor location and physical activity features were found to be highly correlated with the outcomes (community vs hospital re-admission) at baseline. In this section, we discuss the significant highlights of the result.

### Steps Versus Raw Acceleration Signal

Interestingly, walking, a known distinctive parameter in assessing physical functional performance in certain older populations [8], did not yield significance in this study. In populations that are frail, similar to that in subacute rehabilitation, only a negligible amount of time is spent walking (<1% of daily activity). This suggests that in these populations, steps counters may not necessarily be the best way to quantify active state [24,25]. It would be best to prepare for the stark reality that geriatric population may not be active enough to assess their well-being or infer their independence only based on step counts or by monitoring their walking. A combination of activity features that includes both wearable sensor and stationary beacons that provide corresponding indoor localizations could be a stronger indicator of their general well-being and/or frailty. Moreover, the use of raw acceleration signals to quantify energy intensity allows us to capture even small movements, the movements that may not trigger step counters but still indicate some level of activity. Let us consider an example in which we considered energy spent rather than steps: compared with community, hospital patients show higher percentage of energy while laying down (*P*=.39, *d*=0.54). They also spent more overall time (%) in that position (60.99) compared with 46.83 for community patients. However, energy intensity of community patients is higher than hospital patients (26.23 vs 19.54). This indicates that community patients have been more active while lying down. Being more active while lying down may be the result of turning in bed; hence, this feature may denote higher ability to move in community patients. In this scenario, as discussed earlier, step counters will not produce reliable results to quantify patients’ activity levels.

### Activity With Therapist Versus Resident Time Alone

One interesting aspect of this study was to investigate the activity while a patient is with a physical therapist versus activity during the other hours of the day. It did not appear that a clear distinction could be made between different outcome groups based on therapy room energy intensity. This could be because all patients during therapy sessions are engaged by the therapist in similar physical activities following set protocols. However, the energy intensity of resident room was distinctive within outcome groups.

### Value of Indoor Localization Data

To assess the value of indoor localization in activity tracking, it would be best to highlight some of the scenarios: according to Table 4, among clinical characteristic assessment, ADL toilet (*P*=.007) was the most significant feature in determining the outcome. This feature corresponds to the watch-derived feature energy intensity in resident bathroom. With *P*=.004 and effect size of *d*=1.18, energy intensity in *resident room* (achieved from indoor localization) hence confirms the clinical finding and can be considered in the absence of ADL evaluations. In other words, ADL variant, a highly significant clinical feature, can be replicated using combination of indoor localization and activity/energy derivations.

Both group energy intensities at bed and bath were less than 60 per min. In the study by Razjouyan et al [8], authors use a cutoff point of 90 to differentiate between light and moderate-to-vigorous activities. On the basis of that, given the intensity in both bathroom and bed for either of the groups, we can conclude that patients performed light activities in those locations.

None of the patients in hospital outcome group used therapy room toilet/bathroom. It is likely that those patients were not capable enough to perform such exercises or even not advised by clinicians/nurses to do so to prevent injury. Either way, the lack of performing an activity, in this case, information extracted from indoor localization data, could be an early indication of which group a patient belongs to; it could also potentially be used to identify adverse outcomes and proactively address to prevent a negative outcome.

### Predictive Analysis: Statistically Significant Features

*P* value as statistical significance or strength of evidence index has long been a subject of debate [31,32]. It is very crucial to know that the *P* value is not a definite test; increasing more attributes significantly correlated with the outcome variable in a predictive model does not necessarily yield higher predictability. Although statistical significance index and its effect size provide a standard exploratory data analysis and perhaps a good informal heuristic for choosing attributes of a prediction model, machine learning practice has more freedom from model assumptions. This study shows that the addition of significant variants did not increase predictive power and the model with only energy intensity in resident room produced the highest recall of minority class (hospital outcome) and overall AUC (0.84).

Considering only the prediction results, we can infer that location data add value to our system. It is apparent that energy intensity in resident room is the most decisive feature in predicting the outcome.

### Limitations and Future Research

Activity classification can best be obtained using a series of motion sensors placed on various parts of the body. Thus, a wide range of activities can be captured as most body motions are detected. However, to simplify the activity detection, using single motion sensors is quite popular. Placing an accelerometer on the hip has been one of the most popular methods because it captures almost all human motions; however, it underestimates the arm ergometry, as it cannot fully extract the arm movements [33]. Wrist-worn accelerometers are popular because of their ease of use, water resistance in most brands, and capturing a comprehensive set of activities. However, interpreting their data for certain sedentary activities such as sitting, standing, and laying is rather challenging, in that, hand movements are very similar in those scenarios. Although ambulation detection is evident in most cases, error rates of classification increase when using assistive devices, walking in very low speed, carrying a weight with the hand that is not wearing the watch, or doing activities involving hand and feet movement together such as sweeping [15,33,34].

Patients’ compliance with wearing a smartwatch was the main challenge of this study, and we expect it to be a generic obstacle in similar studies that aim to harness wearable technology for patients. Moreover, if the target population is less familiar with new forms of technology such as wearable devices, the compliance issue might become even more crucial. In this study, we recruited 184 patients, of which 30 patients were excluded for not satisfying the analysis inclusion criteria (watch wear time constraint). Our baseline analyses revealed that 50% of patients removed their watches before the study coordinator collects them at the end of the 8 hours.

Dealing with medical datasets is rather challenging in that the datasets predominantly consist of normal cases in addition to minority abnormal instances that deem to be more interesting [35]. Many attempts have been made to overcome the obstacle of the normal and abnormal samples known as imbalanced datasets. There exist approaches to improve the performance of predictive models by oversampling and/or undersampling the dominant and abnormal instances [36-38]. In our study cohort, the 2 outcome categories are not equally represented, making the dataset imbalanced. In the future, we aim to further investigate the use of oversampling and undersampling of our dataset as methods that perhaps are not very conventional in the medical field but can possibly improve the predictability of our models.

Next step would be the longitudinal analysis on the same study cohort over the 21-day period they were admitted to the same rehabilitation center. This will allow us to track the trends in sensor feature values and investigate if their changes mimic the daily assessment change performed by clinicians. The result can allow development of models of early frailty detection or producing intervention alerts.

### Conclusions

Despite the evolution of eHealth and mobile health (mHealth) and the emerging role of wearable and mobile technology in new platforms of health care, there are anecdotal claims that wearable technology may not precisely quantify patients’ health [39]. In this study, we showed that wearable technology, equipped with refined physical activity tracking algorithms, in our case, tailored for geriatrics, can result in a better understanding of patients and hopefully pave the way in developing intervention alerts and approaches. We discussed how SARP features provide a clearer storyline of daily activity patterns by merging indoor localization with physical activities. The SARP system can be incorporated into mHealth technology platforms and can provide a more objective assessment of the frail population.

## Abbreviations

ADL: activity of daily living
AUC: area under the curve
BLE beacon: bluetooth low-energy sensors
C: community
H: hospital
KDE: kernel density estimation
MAD: mean absolute deviation
mHealth: mobile health
RSSI: Received Signal Strength Indicator
SARP: Sensing At-Risk Population
SM: signal magnitude
